# Comparative efficacy of six therapies for Hypopharyngeal and laryngeal neoplasms: a network meta-analysis

**DOI:** 10.1186/s12885-019-5412-z

**Published:** 2019-03-29

**Authors:** Juan Che, Yanlin Wang, Xiaolin Zhang, Jun Chen

**Affiliations:** Department of Otorhinolaryngology, Binzhou Medical University Hospital, No. 661 Second Huanghe Road, Binzhou, 256603 Shandong China

**Keywords:** Hypopharyngeal neoplasm, Laryngeal neoplasm, Efficacy, Network meta-analysis

## Abstract

**Background:**

Hypopharyngeal and laryngeal neoplasms are both fatal and hard to catch in early stages. Yet which treatment is the most efficacious one still remain unanswered. This network meta-analysis (NMA) was conducted to investigate effectiveness of six therapies being utilized in clinical practice nowadays.

**Methods:**

PubMed and Embase were retrieved to synthesize data. Direct and indirect evidence was combined to compare efficacy of treatments. A relative ranking of the six regimens was calculated by the surface under the curve ranking area (SUCRA).

**Results:**

A total of 28 trials with 9109 patients were included in our NMA. Five endpoints investigated included 3/5-year overall survival (3/5-OS), 3/5-year disease free survival (3/5-DFS) and 5-year overall survival rate (5-OSR). In terms of all efficacy outcomes, radiotherapy combined with surgery (RT + S) proved to be better than other therapies while radiotherapy (RT) alone also performed well. Induction chemoradiotherapy (ICRT) was the best regarding 3-DFS (SUCRA = 0.846) while current chemoradiotherapy (CCRT) ranked first in 5-DFS (SUCRA = 0.933) according to SUCRA results. No significant differences were demonstrated in 5-DFS and 5-OSR as shown in the results of NMA.

**Conclusions:**

RT combined with surgery turned out to be optimal therapy of all the outcomes while the efficacy of RT was relatively poorer in the treatment of patients with larynx stage III-IV and hypopharynx stage II-IV. Also, the good performance of CCRT and ICRT in terms of DFS made them as secondary recommended therapies. There is no significant difference between surgery and transoral laser microsurgery (TLM) alone.

**Electronic supplementary material:**

The online version of this article (10.1186/s12885-019-5412-z) contains supplementary material, which is available to authorized users.

## Background

Hypopharyngeal neoplasm, as a term used for malignancies of a subsite of the upper aerodigestive tract, is difficult to catch in its earliest stages. Meanwhile it is reported to have the highest mortality rates among kinds of head and neck cancers [1]. Laryngeal neoplasms are mostly squamous cell carcinomas, which reflect they originate from the skin of the larynx. Its 5 years survival rates in the United States are 60% [2]. So far multiple treatments for hypopharyngeal or laryngeal neoplasms have been developed and compared, which include surgery, radiation therapy (RT), chemoradiation therapy (CRT), or kinds of combination of treatments aforementioned [3]. One of the main targets of treating patients with locoregional advanced laryngeal cancer is to maintain larynx function. Yet surgery, though providing satisfying local control, might impair life quality of patients. Thus the application of surgery alone in treating laryngeal neoplasms and hypopharyngeal neoplasms has been reduced while chemotherapy (CT) and RT have been more widely used in clinical practice these days [2]. However, RT and CT being applied separately or combined have triggered worries regarding a decrease in overall survival rate (OSR) since they are reckoned to have caused acute toxicity and disrupted laryngeal function [4].

Many meta-analyses related to head and neck cancer, hypopharyngeal neoplasm, and laryngeal neoplasms have been conducted to evaluate the efficacy of CT, RT, and CRT. Whereas, there were several deficiencies in prior studies such as small sample sizes [5] or a lack of sufficient prospective studies [5]. Besides, the nature of conventional meta-analysis prevents it from performing more comprehensive analysis of multiple treatments. It is known that network meta-analysis (NMA) could combine both direct and indirect evidence, thus considered to be of high clinical value. Based on the facts mentioned above, NMA might be the most adequate way to compare different regimens of hypopharyngeal neoplasms and laryngeal neoplasms.

Notably, most of existing NMAs comparing treatments for head and neck cancers failed to pay attention to certain specific types of cancers, which are hypopharyngeal and laryngeal neoplasms in our case. Hence it is necessary to conduct a systematic review in order to get more comprehensive results regarding regimens for hypopharyngeal and laryngeal neoplasms.

## Methods

### Research reporting format

This NMA was conducted according to the preferred reporting items for systematic reviews and meta-analyses (PRISMA) guidelines.

### Search strategy

PubMed and Embase were systematically retrieved for eligible trials. Subject terms and key words including “hypopharyngeal neoplasms”, “laryngeal neoplasms”, “radiotherapy”, “chemoradiotherapy”, “transoral laser microsurgery”, “radiotherapy combined with surgery”, “induction chemotherapy radiotherapy”, “current chemotherapy radiotherapy”, and “clinical controlled trials” were searched with Boolean operators OR and AND. Titles and abstracts of all studies obtained after searching were reviewed in turn to screen their qualifications.

### Inclusion and exclusion criteria

Included studies were restricted by the following criteria: (1) the study was clinical controlled trial; (2) patients were diagnosed with hypopharyngeal neoplasms or laryngeal neoplasms; (3) therapies included at least one of the six following treatments: surgery, RT, transoral laser microsurgery (TLM), radiotherapy combined with surgery (RT + S), induction chemotherapy radiotherapy (ICRT), and current chemotherapy radiotherapy (CCRT); (4) at least one of the following relevant outcomes was provided: 3/5-year overall survival (3/5-OS), 3/5-year disease free survival (3/5-DFS) and 5-year overall survival rate (5-OSR). Conference abstracts, reviews, duplicates and articles lacking sufficient information or relevant outcomes were excluded. Two experienced reviewers performed an assessment independently to examine eligibility. Any possible disagreements between them would be resolved by consensus and still remaining disagreements would be referred to a third reviewer.

### Outcome measurement and data extraction

Baseline characteristics of patients and basic information including first author, year of publication, blind test, etiology, follow-up duration, intervention, and sample size of each treatment were extracted by two reviewers from eligible trials. Discrepancies were solved by discussion or a third reviewer. Five outcomes were selected in order to assess the efficacy of surgery and other five regimens.

### Statistical analysis

This NMA is conducted by R software (Version 3.1.3) and STATA (Version 13.0). A random-effect Bayesian model was utilized considering the existence of heterogeneity. Network plots demonstrated all efficacy outcomes of six regimens. Hazard ratios (HR) with 95% credible intervals (CrIs) were calculated to perform a NMA, combining both direct and indirect evidence. The results of NMA were shown in forest plots and heat plots were utilized in our NMA to evaluate the inconsistency, in which colors were associated with the change in inconsistency between direct and indirect evidence (shown in the row) after detaching the effect of certain evidence (shown in the column). In heat plots, cold colors indicated an increase of consistency while warm colors indicated a decrease of consistency. In addition, relative efficacy of six therapies was ranked based on surface under the cumulative ranking curve (SUCRA). Higher probabilities revealed in SUCRA signified more desirable interventions.

### Subgroup analysis

A subgroup analysis for locally advanced hypopharyngeal neoplasms or laryngeal neoplasms patients were conducted for the existence of clinical heterogeneity between studies. The subgroup analysis involved 17 studies reported patients with larynx stage III-IV and hypopharynx stage II-IV only.

## Results

### Literature search

A total of 4737 potential relevant publications were identified by literature search. Four thousand five hundred eighty-three remained for screening after duplicates were deleted. Four thousand three hundred forty-eight articles were excluded due to irrelevant titles and abstracts. Among the 235 full-text studies being assessed, 207 papers were removed on account of insufficient data, single arm studies, and irrelevant outcomes. Finally, 28 trials met the inclusion criteria and were retrieved for data extraction [2, 3, 6–31].

**Fig. 1:**
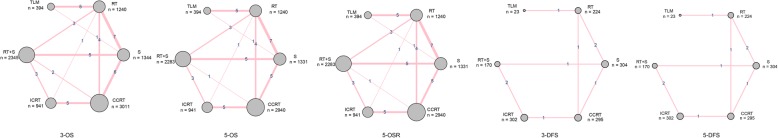
The network of comparisons of 3-OS, 5-OS, 3-DFS, and 5-DFS. (The width of the lines is proportional to the number of trials comparing each pair of treatments; the area of circles represents the cumulative number of patients for each intervention.) S, surgery; RT, radiotherapy; TLM, transoral laser microsurgery; RT + S, surgery combined with radiotherapy; ICRT, Induction chemotherapy radiotherapy; CCRT, current chemotherapy radiotherapy

Table 1 and Additional file 1: Table S1 summarized basic information of included trials and patients characteristics. Among 28 trials with 9109 patients, most were two-arm trials comparing one kind of treatment with surgery or a surgery combined with other regimens. Among the included studies, there were seven trials designed to be randomized controlled trials while 21 were reported as retrospective cohort studies. The networks of comparisons between different treatments with corresponding sample sizes were summarized in Fig. 1. Each circle indicated a certain intervention while the size of each circle denoted its sample size. The thickness of lines linking two circles signified the amount of literatures comparing the corresponding two therapies.

**Table 1:** Basic characteristics of included studies

Study	Design	Cancer type	Stage	No. of patients	Comparison
Wolf, 2017	Retrospective	Glottic/supraglottic cancer	I-IV	247	RT vs. CCRT vs. S
Stokes, 2017	Retrospective	Larynx cancer	IV	3542	RT + S vs. CCRT vs. ICRT
Low, 2017	Retrospective	Laryngeal SCC	I	105	TLM vs. RT
Peng, 2016	Retrospective	Glottic cancer	I-II	172	RT vs. S
Marchiano, 2016	Retrospective	Subglottic SCC	I-IV	576	RT vs. S vs. RT + S
De Santis, 2016	Retrospective	Glottic cancer	I-II	75	TLM vs. RT
Timmermans, 2015	Retrospective	Laryngeal cancer	III-IV	182	S vs. CCRT vs. RT
Timme, 2015	Retrospective	Laryngeal cancer	III-IV	71	S vs. CCRT
Li, 2015	Retrospective	Laryngeal cancer	I-IV	309	RT vs. S vs. RT + S
Grover, 2015	Retrospective	Larynx cancer	IV	969	S vs. CCRT
Hsin, 2014	Retrospective	Laryngeal cancer	IV	62	S vs. CCRT
Lefebvre, 2013	Randomized	Larynx/Hypopharynx SCC	II-IV	118	CCRT vs. ICRT
Forastiere, 2013	Randomized	Larynx cancer	III-IV	520	CCRT vs. ICRT vs. RT
Lefebvre, 2012	Randomized	Hypopharyngeal SCC	II-IV	194	RT + S vs. ICRT
Patel, 2011	Retrospective	Larynx cancer	IV	34	CCRT vs. S
Mahler, 2010	Retrospective	Glottic cancer	I	351	RT vs. TLM
Dinapoli, 2010	Retrospective	Glottic cancer	I-II	143	RT vs. S
Schrijvers, 2009	Retrospective	Glottic laryngeal cancer	I	100	RT vs. TLM
Thurnher, 2008	Retrospective	Glottic laryngeal SCC	I	337	S vs. TLM vs. RT
Boscolo-Rizzo, 2008	Retrospective	Laryngeal cancer	III-IV	112	RT + S vs. CCRT
Andreadis, 2007	Retrospective	Laryngeal cancer	III-IV	50	ICRT vs. CCRT
Bensadoun, 2006	Randomized	Laryngeal cancer	III-IV	163	RT vs. CCRT
Richard, 1998	Randomized	Laryngeal cancer	III-IV	68	RT + S vs. ICRT
Beauvillain, 1997	Randomized	Hypopharyngeal cancer	III-IV	90	RT + S vs. S
Bryant, 1995	Retrospective	Glottic cancer	III	97	RT vs. RT + S
Frank, 1994	Retrospective	Hypopharyngeal SCC	I-IV	109	S vs. RT + S
Jones, 1992	Retrospective	Larynx cancer	III-IV	147	S vs. RT + S
Wolf, 1991	Randomized	Larynx cancer	III-IV	166	ICRT vs. CCRT

Abbreviation: *S* surgery, *RT* radiotherapy, *TLM* transoral laser microsurgery, *RT + S* surgery combined with radiotherapy, *ICRT* Induction chemotherapy radiotherapy, *CCRT*, current chemotherapy radiotherapy

### Outcomes

**Fig. 2:**
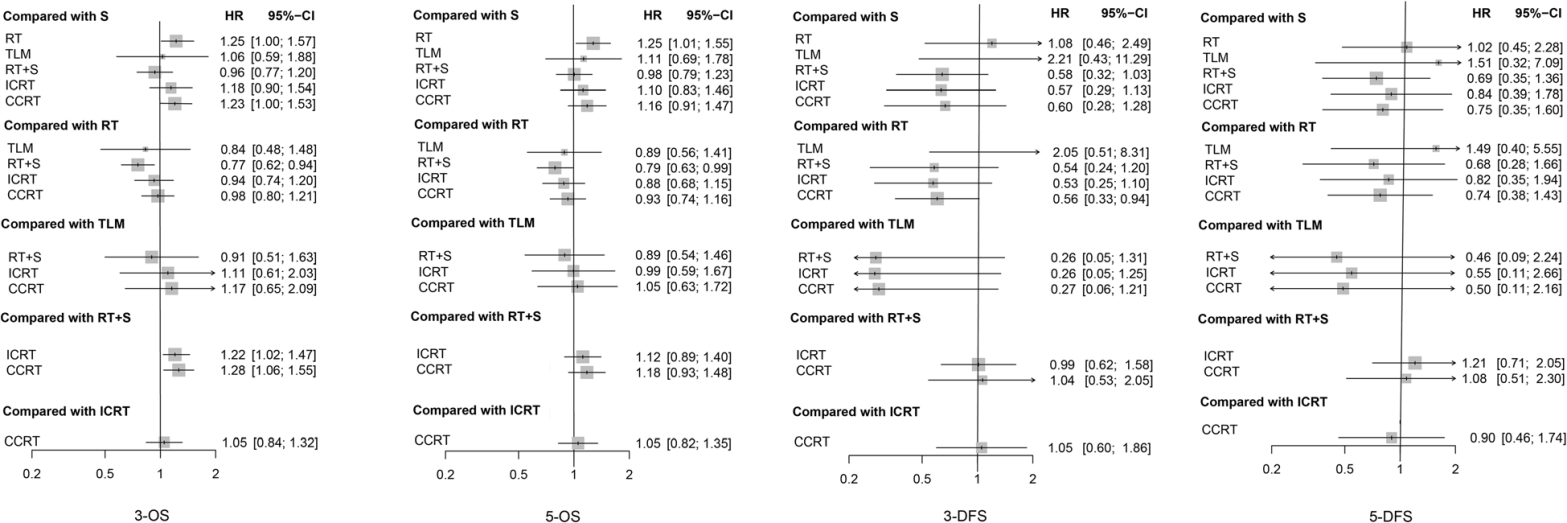
Forest plots for four endpoints. Hazard ratio (HRs) and 95% credible interval (CrIs) indicate the relative efficacy under the corresponding endpoint. S, surgery; RT, radiotherapy; TLM, transoral laser microsurgery; RT + S, surgery combined with radiotherapy; ICRT, Induction chemotherapy radiotherapy; CCRT, current chemotherapy radiotherapy

In this NMA, 3-OS, 5-OS, 3-DFS, 5-DFS, and 5-OSR were calculated as primary outcomes, the results of which were demonstrated in Table 2 and Fig. 2.

**Table 2:** Network meta-analysis results with hazard ratio (HR) for 3-OS, 5-OS, 3-DFS, and 5-DFS, and odds ratio (OR) for 5-OSR

3-OS					
S	0.8 (0.64, 1.00)	0.95 (0.95, 1.68)	1.04 (0.83, 0.30)	0.85 (0.65, 1.11)	0.81 (0.65, 1)
**1.25 (1.00, 1.57)**	RT	1.19 (0.68, 2.08)	1.31 (1.06, 1.61)	1.07 (0.84, 1.36)	1.02 (0.83, 1.24)
1.06 (0.59, 1.88)	0.84 (0.48, 1.48)	TLM	1.1 (0.61, 1.98)	0.90 (0.49, 1.64)	0.86 (0.48, 1.53)
0.96 (0.77, 1.20)	**0.77 (0.62, 0.94)**	0.91 (0.51, 1.63)	RT + S	0.82 (0.68, 0.98)	0.78 (0.64, 0.94)
1.18 (0.90, 1.54)	0.94 (0.74, 1.20)	1.11 (0.61, 2.03)	**1.22 (1.02, 1.47)**	ICRT	0.95 (0.76, 1.20)
**1.23 (1.00, 1.53)**	0.98 (0.80, 1.21)	1.17 (0.62, 2.09)	**1.28 (1.06, 1.55)**	1.05 (0.84, 1.32)	CCRT
5-OS					
S	0.80 (0.64, 0.99)	0.90 (0.56, 1.45)	1.02 (0.81, 1.27	0.91 (0.69, 1.21)	0.86 (0.68, 1.10)
**1.25 (1.01, 1.55)**	RT	1.13 (0.71, 1.80)	1.27 (1.01, 1.59)	1.14 (0.87, 1.48)	1.08 (0.86, 1.35)
1.11 (0.69, 1.78)	0.89 (0.56, 1.41)	TLM	1.12 (0.68, 1.85)	1.01 (0.60, 1.69	0.96 (0.58, 1.58)
0.98 (0.79, 1.23)	**0.79 (0.63, 0.99)**	0.89 (0.54, 1.46)	RT + S	0.90 (0.71, 1.13)	0.85 (0.68, 1.07)
1.10 (0.83, 1.46)	0.88 (0.68, 1.15)	0..99 (0.59, 1.67)	1.12 (0.89, 1.40)	ICRT	0.95 (0.74, 1.22)
1.16 (0.91, 1.47)	0.93 (0.74, 1.16)	1.05 (0.63, 1.72)	1.18 (0.93, 1.48)	1.05 (0.82, 1.35)	CCRT
3-DFS					
S	0.93 (0.40, 2.15)	0.45 (0.09, 2.32)	1.73 (0.98, 3.08)	1.76 (0.89, 3.48)	1.67 (0.78, 3.57)
1.08 (0.46, 2.49)	RT	0.49 (0.12, 1.98)	1.86 (0.84, 4.16)	1.89 (0.91, 3.95)	1.79 (1.06, 3.04)
2.21 (0.43, 11.29)	2.05 (0.51, 8.31)	TLM	3.82 (0.76, 19.20)	3.88 (0.80, 18.84)	3.68 (0.82, 16.42)
0.58 (0.32, 1.03)	0.54 (0.32, 1.03)	0.26 (0.05, 1.31)	RT + S	1.01 (0.63, 1.62)	0.96 (0.49, 1.90)
0.57 (0.29, 1.13)	0.53 (0.25, 1.1)	0.26 (0.05, 1.25)	0.99 (0.62, 1.58)	ICRT	0.95 (0.54, 1.67)
0.6 (0.28, 1.28)	**0.56 (0.33, 0.94)**	0.27 (0.06, 1.21)	1.04 (0.53, 2.05)	1.05 (0.60, 1.86)	CCRT
5-DFS					
S	0.98 (0.44, 2.21)	0.66 (0.14, 3.09)	1.44 (0.73, 2.83)	1.20 (0.56, 2.55)	1.33 (0.63, 2.82)
1.02 (0.45, 2.28)	RT	0.67 (0.18, 2.5)	1.46 (0.60, 3.56)	1.21 (0.51, 2.87)	1.35 (0.70, 2.60)
1.51 (0.32, 7.09)	1.49 (0.40, 5.55)	TLM	2.18 (0.45, 10.67)	1.81 (0.38, 8.7)	2.01 (0.46, 8.74)
0.69 (0.35, 1.36)	0.68 (0.28, 1.66)	0.46 (0.09, 2.24)	RT + S	0.83 (0.49, 1.41)	0.92 (0.43, 1.96)
0.84 (0.39, 1.78)	0.82 (0.35, 1.94)	0.55 (0.11, 2.66)	1.21 (0.71, 2.05)	ICRT	1.11 (0.57, 2.16)
0.75 (0.35, 1.60)	0.74 (0.38, 1.43)	0.5 (0.11, 2.16)	1.08 (0.51, 2.30)	0.9 (0.46, 1.74)	CCRT
5-OSR					
S	0.78 (0.31, 1.82)	2.08 (0.53, 7.33)	1.28 (0.52, 3.08)	0.94 (0.34, 2.63)	0.93 (0.39, 2.19)
1.29 (0.55, 3.24)	RT	2.71 (0.84, 8.16)	1.66 (0.61, 4.68)	1.22 (0.45, 3.47)	1.20 (0.51, 2.99)
0.48 (0.14, 1.87)	0.37 (0.12, 1.20)	TLM	0.61 (0.15, 2.79)	0.45 (0.11, 2.14)	0.44 (0.12, 1.84)
0.78 (0.32, 1.91)	0.60 (0.21, 1.64)	1.64 (0.36, 6.57)	RT + S	0.73 (0.28, 1.89)	0.73 (0.28, 1.85)
1.06 (0.38, 2.97)	0.82 (0.29, 2.24)	2.24 (0.47, 9.15)	1.37 (0.53, 3.55)	ICRT	0.99 (0.44, 2.18)
1.07 (0.46, 2.57)	0.83 (0.33, 1.95)	2.25 (0.54, 8.40)	1.38 (0.54, 3.57)	1.01 (0.46, 2.28)	CCRT

Note: Figures in bold font show significant outcome

Abbreviation: *S* surgery, *RT* radiotherapy, *TLM* transoral laser microsurgery, *RT + S* surgery combined with radiotherapy, *ICRT* Induction chemotherapy radiotherapy, *CCRT* current chemotherapy radiotherapy, *3-OS* 3-year overall survival, *5-OS* 5-year overall survival, *3-DFS* 3-year disease free survival, *5-DFS* 5-year disease free survival;5-OSR, 5-year overall survival rate

In terms of 3-OS, surgery alone performed significantly better than RT and CCRT (RT: HR 1.25, 95% CrI 1.00–1.57; CCRT: HR 1.23, 95% CrI 1.00–1.53). Meanwhile, RT combined with surgery turned out to be significantly superior to RT, ICRT, and CCRT (RT: HR 1.31, 95% CrI 1.06–1.61; ICRT: HR 1.22, 95% CrI 1.02–1.47; CCRT: HR 1.28, 95% CrI 1.06–1.55). With regard to 5-OS, RT combined with surgery and surgery alone both had significant advantage over RT (HR 1.25, 95% CrI 1.01–1.55; HR 1.27, 95% CrI 1.01–1.59, respectively). Apart from that, there existed no other significant difference among comparisons of 6 regimens.

The results of 3-DFS were in favor of CCRT, since CCRT proved to be more efficacious than RT with statistical significance (HR 1.79, 95% CrI 1.06–3.04). Yet no other significant difference occurred regarding 3-DFS. Notably, there also existed no significant difference among comparisons in 5-DFS and 5-OSR.

### Relative ranking analysis

A relative ranking of the 6 therapies could be obtained on the basis of SUCRA results (Table 3). RT combined with surgery and surgery alone demonstrated desirable performance. RT combined with surgery and surgery alone ranked first and second respectively in both 3-OS (RT + S: 0.878; S: 0.763) and 5-OS (RT + S: 0.930; S: 0.773). Besides, surgery ranked highest in 5-ORS (0.716) while RT combined with surgery rank the third place in 3-DFS (0.717).

**Table 3:** Surface under the cumulative ranking curve (SUCRA) for the treatments under five endpoints

Treatments	3-OS	5-OS	3-DFS	5-DFS	5-OSR
S	0.763	0.773	0.257	0.125	0.716
RT	0.243	0.151	0.291	0.457	0.076
TLM	0.582	0.486	0.088	0.244	0.687
RT + S	0.878	0.930	0.717	0.600	0.621
ICRT	0.244	0.248	0.846	0.639	0.413
CCRT	0.287	0.410	0.798	0.933	0.484

Abbreviation: *S* surgery, *RT* radiotherapy, *TLM* transoral laser microsurgery, *RT + S* surgery combined with radiotherapy, *ICRT* Induction chemotherapy radiotherapy, *CCRT* current chemotherapy radiotherapy, *3-OS* 3-year overall survival, *5-OS* 5-year overall survival, *3-DFS* 3-year disease free survival, *5-DFS* 5-year disease free survival, *5-OSR* 5-year overall survival rate

Moreover, ICRT and CCRT proved to be advantageous regarding DFS. ICRT was the optimal therapy in 3-DFS (0.846) and suboptimal in 5-DFS (0.639) while CCRT turned out to be next-best in 3-DFS (0.798) and the best in 5-DFS (0.933).

### Inconsistency assessment

**Fig. 3:**
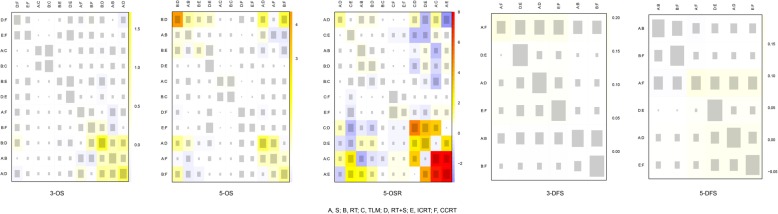
The results of consistency analysis by heat plot for each outcome. The size of the gray squares indicates the contribution of the direct evidence (shown in the column) to the network evidence (shown in the row). The colors are associated with the change in inconsistency between direct and indirect evidence (shown in the row) and the warmer color represents the stronger inconsistency. S, surgery; RT, radiotherapy; TLM, transoral laser microsurgery; RT + S, surgery combined with radiotherapy; ICRT, Induction chemotherapy radiotherapy; CCRT, current chemotherapy radiotherapy

As shown in heat plots (Fig. 3), there is no evidence of inconsistency existing in the direct and indirect evidence in 3-OS, 5-OS, 3-DFS, and 5-DFS. Yet there was a sign of inconsistency regarding 5-OSR according to the results of heat plot.

### Subgroup analysis

Among 28 included studies, 7 papers included only early-stage patients, 4 papers included both early-stage and locally advanced stages patients and 17 papers only included locally advanced stages patients (larynx stage III-IV and hypopharynx stage II-IV). For the treatments related to 7 papers included only early-stage patients were limited and the data were not sufficient enough to draw reliable conclusion, we decided to add a subgroup analysis of 17 papers only included locally advanced stages patients (larynx stage III-IV and hypopharynx stage II-IV). The network meta-analysis results were shown in Additional file 2: Table S2 and SUCRA results were shown in Additional file 3: Table S3. Surgery, RT, RT + S, ICRT, and CCRT were involved in subgroup analysis. In term of 3-OS and 5-OS, RT + S showed a relatively lower HR and better performance when compared with others without statistically significance; in term of 3-DFS and 5-DFS, CCRT and surgery alone had similar performance. In term of 5-OSR, RT + S and surgery alone performed significantly better than RT (HR 0.39, 95% CrI 0.17–0.9; HR 0.31, 95% CrI 0.13–0.74). The SUCRA results shown in Additional file 3: Table S3 that RT + S was the probably best treatments as for 3-OS and 5-OS (SUCRA = 0.840, 0.808). CCRT may be the best treatments in increasing 3-DFS (0.695) while ICRT may be the best treatments in increasing 5-DFS (0.771). Surgery alone ranked the highest in 5-OSR (0.337).

## Discussion

Our NMA focused on comparative efficacies of six therapies of hypopharyngeal neoplasms and laryngeal neoplasms. Results of NMA indicated that surgery alone were significantly better than RT and CCRT according to 1-OS outcome. RT + S were significantly superior to RT, ICRT, and CCRT in terms of 3-OS. Meanwhile RT + S and surgery alone had significant advantage over RT in 5-OS. CCRT proved to be better than RT in 3-DFS. With regards to relative ranking analysis, RT + S performed desirably in both OS and DFS while CCRT and ICRT were advantageous in DFS.

RT is utilized as the primary treatment for early laryngeal cancer in northern Europe and North America for its being an acknowledged treatment for selected laryngeal carcinomas that educes beneficial oncologic results [32]. For instance, RT could provide a possibility of curing un-resectable tumor. When compared with surgery, RT might provide less morbidity regarding subclinical disease and advantageous organ preservation [33].

Yet RT performed poorly in both OS and DFS according to our results, which is contrary to the results of a prior NMA claiming that RT was associated with higher rates of OS and DFS than CT, CRT, and laryngectomy [32]. However, our results that CCRT and ICRT took advantageous over RT turned out to be consistent with paper conducted by Zackrisson et al., which concluded that CRT was better than RT [34]. Besides, TLM has been used in laryngeal malignant lesions since 1972. As pointed out in prior NMAs, there are controversies in clinical literature regarding oncologic outcomes of TLM and RT for T1 glottis carcinoma, and plenty of comparisons between TLM and RT have been made. RT was inferior to TLM for T1 glottic carcinoma in OS according to Mo et al. [35], Zhong et al. [36], and Huang et al. [5]. Whereas Abdurehim et al. thought this question remained unanswered in systematic review since no significant difference was shown between RT and TLM [37], which is also consistent with our results. More random-controlled and prospective trials should be conducted, based on which a more clinically reliable conclusion would be reached in future NMAs.

Furthermore, CCRT and ICRT demonstrated no significant difference on survival control in our analysis. Similar results were shown in Ma et al. paper which claimed that ICRT had no significant benefit compared to CCRT regarding OS or locoreginal control [38]. Yet Ma also claimed that ICRT generated desirable outcomes in larynx preservation [39]. More outcomes should be explored with regard to the effectiveness of ICRT and CCRT. The subgroup analysis of 17 studies reported patients with larynx stage III-IV and hypopharynx stage II-IV also showed similar results in term of 3-OS, 5-OS, 3-DFS, 5-DFS except that the results were all not statistically significant. More clinical researches will be required in the future.

There still exist several limitations in our NMA despite that we have conducted our work as meticulous as possible. First of all, there might be several deficiencies caused by the quality and non-unified standards of the included trials. For instance, patients’ or surgeon’s preference in these trials might lead to selection bias inevitably. Besides, a great proportion of the included trials were non-randomized or retrospective trials, which could increase heterogeneity of our NMA. Notably, toxicity of treatments analyzed in our NMA has not been investigated thoroughly because of a lack of data and comparable outcomes. It should not be ignored that a lack of full consideration on dose of RT and type of TLM might undermine the clinical value of our results to some extent. Secondly, we failed to take more combination therapies into consideration. Several trials and conventional meta-analyses have discovered an increase in efficacy of combination therapies such as chemotherapy combined with CCRT on survival control for some types of head and neck cancers [40–42]. Yet a comprehensive analysis regarding various combination treatments is hard to conduct on account of insufficient data in our included studies. Thirdly, sample sizes for some specific comparisons were not big enough. For instance, there were only 23 subjects treated by TLM in 3-DFS and 5-DFS. More prospective trials should be performed to reach more reliable conclusions and further studies focusing on combination therapies should be carried out. Fourthly, due to limited studies and insufficient data on early-stage patients, our analysis could not make a subgroup analysis of these patients. Therefore, future studies are needed to assess the impact of various treatments in the management of early-stage glottic larynx patients in order to provide more credible medical guideline.

## Conclusions

RT combined with surgery turned out to be optimal therapy of all the outcomes while the efficacy of RT was relatively poorer in the treatment of patients with larynx stage III-IV and hypopharynx stage II-IV. Also, the good performance of CCRT and ICRT in terms of DFS made them as secondary recommended therapies. There is no significant difference between surgery and TLM. The comparison of more related therapies will be better understood once results of more high-quality clinical trials conducted.

## Additional files

Additional file 1: **Table S1.** Basic characteristics and endpoints of included studies. (DOCX 39 kb)

Additional file 2: **Table S2.** Network meta-analysis results of subgroup analysis for locally advanced hypopharyngeal and laryngeal neoplasms. (DOCX 19 kb)

Additional file 3: **Table S3.** SUCRA results of subgroup analysis for locally advanced hypopharyngeal and laryngeal neoplasms. (DOCX 16 kb)

## Abbreviations

3/5-DFS: 3/5-year disease free survival
3/5-OS: 3/5-year overall survival
5-OSR: 5-year overall survival rate
CCRT: Current chemoradiotherapy
CrIs: Credible intervals
CRT: Chemoradiation therapy
HR: Hazard ratios
ICRT: Induction chemoradiotherapy
NMA: Network meta-analysis
PRISMA: Preferred reporting items for systematic reviews and meta-analyses
RT + S: Radiotherapy combined with surgery
RT: Radiotherapy
SUCRA: surface under the curve ranking area
TLM: transoral laser microsurgery
