# Supplementary material for: Comparative efficacy of six therapies for Hypopharyngeal and laryngeal neoplasms: a network meta-analysis
Source: BMC Cancer. 2019 Mar 29;19:282. doi: 10.1186/s12885-019-5412-z (PMC6439970; doi:10.1186/s12885-019-5412-z)
Supplement: Supplementary file 1 — Table S1. Basic characteristics and endpoints of included studies. (DOCX 39 kb) [file 12885_2019_5412_MOESM1_ESM.docx]

**Table S1. Basic characteristics and** **endpoints of included studies**

| **Study** | **Design** | **Cancer type** | **Stage** | **No. of patients** | **Comparison** | **3-OS**  **HR** | **3-OS**  **LL** | **3-OS**  **UL** | **5-OS**  **HR** | **5-OS**  **LL** | **5-OS**  **UL** | **5-OSR** | **3-DFS**  **HR** | **3-DFS**  **LL** | **3-DFS**  **UL** | **5-DFS**  **HR** | **5-DFS**  **LL** | **5-DFS**  **UL** |
| --- | --- | --- | --- | --- | --- | --- | --- | --- | --- | --- | --- | --- | --- | --- | --- | --- | --- | --- |
| Wolf , 2017 | Retrospective | Glottic/supraglottic cancer | I-IV | 247 | RT |  |  |  |  |  |  | 0.13 |  |  |  |  |  |  |
|  |  |  |  |  | CCRT | 0.54 | 0.19 | 1.53 | 0.68 | 0.29 | 1.62 | 0.32 |  |  |  |  |  |  |
| Stokes , 2017 | Retrospective | Larynx cancer | IV | 3542 | RT+S | 0.61 | 0.13 | 2.90 | 0.47 | 0.12 | 1.85 | 0.30 |  |  |  |  |  |  |
|  |  |  |  |  | CCRT |  |  |  |  |  |  | 0.23 |  |  |  |  |  |  |
|  |  |  |  |  | ICRT | 1.33 | 1.11 | 1.59 | 1.27 | 1.07 | 1.51 | 0.15 |  |  |  |  |  |  |
| Low , 2017 | Retrospective | Laryngeal SCC | I | 105 | ICRT | 1.44 | 1.27 | 1.63 | 1.46 | 1.32 | 1.62 | 0.20 | 0.30 |  |  | 0.60 |  |  |
|  |  |  |  |  | TLM | 0.21 | 0.01 | 22.00 | 0.45 | 0.03 | 7.60 | 0.85 |  | 0.01 | 300.00 |  | 0.01 | 300.00 |
|  |  |  |  |  | RT |  |  |  |  |  |  | 0.81 |  |  |  |  |  |  |
| Peng , 2016 | Retrospective | Glottic cancer | I-II | 172 | RT |  |  |  |  |  |  | 0.80 | 0.14 |  |  | 0.16 |  |  |
|  |  |  |  |  | S | 0.35 | 0.01 | 121.00 | 0.22 | 0.04 | 1.41 | 0.91 |  | 0.01 | 2.12 |  | 0.02 | 1.32 |
| Marchiano , 2016 | Retrospective | Subglottic SCC | I-IV | 576 | RT |  |  |  |  |  |  | 0.57 |  |  |  |  |  |  |
|  |  |  |  |  | S | 0.76 | 0.44 | 1.31 | 0.83 | 0.52 | 1.33 | 0.64 |  |  |  |  |  |  |
| De Santis , 2016 | Retrospective | Glottic cancer | I-II | 75 | RT+S | 0.97 | 0.67 | 1.39 | 1.08 | 0.72 | 1.63 | 0.55 | 2.05 |  |  | 1.49 |  |  |
|  |  |  |  |  | TLM | 1.02 | 0.43 | 2.40 | 1.05 | 0.54 | 2.05 | 0.91 |  | 0.54 | 7.71 |  | 0.49 | 4.54 |
|  |  |  |  |  | RT |  |  |  |  |  |  | 0.94 |  |  |  |  |  |  |
| Timmermans , 2015 | Retrospective | Laryngeal cancer | III-IV | 182 | S | 0.82 | 0.42 | 1.62 | 0.90 | 0.53 | 1.53 | 0.53 |  |  |  |  |  |  |
|  |  |  |  |  | CCRT | 2.46 | 0.96 | 6.33 | 1.93 | 0.80 | 4.67 | 0.45 |  |  |  |  |  |  |
| Timme , 2015 | Retrospective | Laryngeal cancer | III-IV | 71 | RT |  |  |  |  |  |  | 0.13 |  |  |  |  |  |  |
|  |  |  |  |  | S |  |  |  |  |  |  | 0.46 |  |  |  |  |  |  |
|  |  |  |  |  | CCRT | 1.33 | 0.33 | 5.39 | 1.66 | 0.62 | 4.44 | 0.44 |  |  |  |  |  |  |
| Li , 2015 | Retrospective | Laryngeal cancer | I-IV | 309 | RT |  |  |  |  |  |  | 0.13 |  |  |  |  |  |  |
|  |  |  |  |  | S | 0.49 | 0.24 | 1.01 | 0.52 | 0.28 | 0.97 | 0.67 |  |  |  |  |  |  |
| Grover , 2015 | Retrospective | Larynx cancer | IV | 969 | RT | 0.87 | 0.45 | 1.70 | 0.92 | 0.51 | 1.67 | 0.38 |  |  |  |  |  |  |
|  |  |  |  |  | S |  |  |  |  |  |  | 0.50 |  |  |  |  |  |  |
|  |  |  |  |  | CCRT | 1.27 | 0.99 | 1.62 | 1.27 | 1.03 | 1.57 | 0.39 |  |  |  |  |  |  |
| Hsin , 2014 | Retrospective | Laryngeal cancer | IV | 62 | S |  |  |  |  |  |  | 0.71 |  |  |  |  |  |  |
|  |  |  |  |  | CCRT | 1.03 | 0.29 | 3.72 | 1.11 | 0.42 | 2.90 | 0.48 | 1.12 | 0.22 | 6.89 | 1.13 | 0.35 | 3.61 |
| Lefebvre , 2013 | Randomized | Larynx/Hypopharynx SCC | II-IV | 118 | CCRT |  |  |  |  |  |  | 0.37 |  |  |  |  |  |  |
|  |  |  |  |  | ICRT | 1.02 | 0.33 | 3.15 | 0.95 | 0.37 | 2.45 | 0.33 |  |  |  |  |  |  |
| Forastiere , 2013 | Randomized | Larynx cancer | III-IV | 520 | CCRT | 1.05 | 0.68 | 1.63 | 0.99 | 0.68 | 1.42 | 0.54 |  |  |  |  |  |  |
|  |  |  |  |  | ICRT | 0.95 | 0.60 | 1.50 | 0.86 | 0.59 | 1.25 | 0.58 |  |  |  |  |  |  |
| Lefebvre , 2012 | Randomized | Hypopharyngeal SCC | II-IV | 194 | RT |  |  |  |  |  |  | 0.54 |  |  |  |  |  |  |
|  |  |  |  |  | RT+S |  |  |  |  |  |  | 0.33 |  |  |  |  |  |  |
|  |  |  |  |  | ICRT | 0.70 | 0.45 | 1.09 | 0.79 | 0.60 | 1.03 | 0.13 | 0.82 | 0.58 | 1.16 | 0.90 | 0.73 | 1.10 |
| Patel , 2011 | Retrospective | Larynx cancer | IV | 34 | CCRT |  |  |  |  |  |  |  |  |  |  |  |  |  |
|  |  |  |  |  | S | 0.33 | 0.06 | 1.91 |  |  |  |  |  |  |  |  |  |  |
| Mahler , 2010 | Retrospective | Glottic cancer | I | 351 | RT | 1.32 | 0.47 | 6.29 | 0.89 | 0.30 | 2.60 | 0.95 |  |  |  |  |  |  |
|  |  |  |  |  | TLM | 1.00 |  |  |  |  |  | 0.91 |  |  |  |  |  |  |
| Dinapoli , 2010 | Retrospective | Glottic cancer | I-II | 143 | RT | 0.38 | 0.05 | 3.18 | 0.65 | 0.24 | 1.72 | 0.83 | 0.55 | 0.11 | 2.77 | 0.59 | 0.20 | 1.77 |
|  |  |  |  |  | S |  |  |  |  |  |  | 0.86 |  |  |  |  |  |  |
| Schrijvers , 2009 | Retrospective | Glottic laryngeal cancer | I | 100 | RT | 1.22 | 0.13 | 11.90 | 1.90 | 0.41 | 8.78 | 0.90 |  |  |  |  |  |  |
|  |  |  |  |  | TLM |  |  |  |  |  |  | 0.88 |  |  |  |  |  |  |
| Thurnher , 2008 | Retrospective | Glottic laryngeal SCC | I | 337 | S |  |  |  |  |  |  | 0.13 |  |  |  |  |  |  |
|  |  |  |  |  | TML | 0.98 | 0.39 | 2.46 | 1.01 | 0.54 | 1.88 | 0.68 |  |  |  |  |  |  |
| Boscolo-Rizzo , 2008 | Retrospective | Laryngeal cancer | III-IV | 112 | RT | 1.60 | 0.77 | 3.33 | 1.57 | 0.96 | 2.56 | 0.13 |  |  |  |  |  |  |
|  |  |  |  |  | RT+S |  |  |  |  |  |  |  |  |  |  |  |  |  |
|  |  |  |  |  | CCRT | 1.33 | 0.06 | 28.88 |  |  |  |  |  |  |  |  |  |  |
| Andreadis , 2007 | Retrospective | Laryngeal cancer | III-IV | 50 | ICRT | 1.16 | 0.30 | 4.48 | 0.92 | 0.58 | 1.48 | 0.43 |  |  |  |  |  |  |
|  |  |  |  |  | CCRT |  |  |  |  |  |  | 0.31 |  |  |  |  |  |  |
| Bensadoun , 2006 | Randomized | Laryngeal cancer | III-IV | 163 | RT |  |  |  |  |  |  | 0.10 |  |  |  |  |  |  |
|  |  |  |  |  | CCRT | 0.68 | 0.46 | 1.01 | 0.67 | 0.49 | 0.92 | 0.23 | 0.56 | 0.40 | 0.79 | 0.76 | 0.60 | 0.95 |
| Richard , 1998 | Randomized | Laryngeal cancer | III-IV | 68 | RT+S |  |  |  |  |  |  | 0.44 | 0.66 | 0.25 | 1.75 | 0.65 | 0.35 | 1.19 |
|  |  |  |  |  | ICRT | 0.84 | 0.24 | 2.89 | 0.76 | 0.38 | 1.55 | 0.58 |  |  |  |  |  |  |
| Beauvillain , 1997 | Randomized | Hypopharyngeal cancer | III-IV | 90 | RT+S |  |  |  |  |  |  | 0.18 |  |  |  |  |  |  |
|  |  |  |  |  | S | 0.72 | 0.35 | 1.46 | 0.75 | 0.50 | 1.12 | 0.37 |  |  |  |  |  |  |
| Bryant , 1995 | Retrospective | Glottic cancer | III | 97 | RT |  |  |  |  |  |  | 0.51 |  |  |  |  |  |  |
|  |  |  |  |  | RT+S | 1.23 | 0.54 | 2.81 | 0.89 | 0.54 | 1.47 | 0.57 |  |  |  |  |  |  |
| Frank , 1994 | Retrospective | Hypopharyngeal SCC | I-IV | 109 | S |  |  |  |  |  |  | 0.28 |  |  |  |  |  |  |
|  |  |  |  |  | RT+S | 0.80 | 0.47 | 1.37 | 0.80 | 0.55 | 1.15 | 0.43 | 0.53 | 0.34 | 0.84 | 0.59 | 0.42 | 0.83 |
| Jones , 1992 | Retrospective | Larynx cancer | III-IV | 147 | S |  |  |  |  |  |  | 0.47 |  |  |  |  |  |  |
|  |  |  |  |  | RT+S | 0.53 | 0.31 | 0.91 | 0.59 | 0.42 | 0.83 | 0.70 |  |  |  |  |  |  |
| Wolf , 1991 | Randomized | Larynx cancer | III-IV | 166 | ICRT |  |  |  |  |  |  | 0.48 |  |  |  |  |  |  |
|  |  |  |  |  | CCRT | 1.12 | 0.76 | 1.65 | 0.91 | 0.70 | 1.19 | 0.59 | 0.97 | 0.63 | 1.51 | 0.77 | 0.57 | 1.03 |

Abbreviation: S, surgery; RT, radiotherapy; TLM, transoral laser microsurgery; RT+S, surgery combined with radiotherapy; ICRT, Induction chemotherapy radiotherapy; CCRT, current chemotherapy radiotherapy; 3-OS, 3-year overall survival; 5-OS, 5-year overall survival; 3-DFS, 3-year disease free survival; 5-DFS, 5-year disease free survival; 5-OSR, 5-year overall survival rate; HR, hazard ratio; LL, lower 95% confidence interval; UL, upper 95% confidence interval.
