# Supplementary material for: Comparative efficacy of six therapies for Hypopharyngeal and laryngeal neoplasms: a network meta-analysis
Source: BMC Cancer. 2019 Mar 29;19:282. doi: 10.1186/s12885-019-5412-z (PMC6439970; doi:10.1186/s12885-019-5412-z)
Supplement: Supplementary file 2 — Table S2. Network meta-analysis results of subgroup analysis for locally advanced hypopharyngeal and laryngeal neoplasms. (DOCX 19 kb) [file 12885_2019_5412_MOESM2_ESM.docx]

**Table S2.** Network meta-analysis results of subgroup analysis for locally advanced hypopharyngeal and laryngeal neoplasms.

|  |  | **CCRT** | **ICRT** | **RT** | **RT+S** | **S** |  |
| --- | --- | --- | --- | --- | --- | --- | --- |
| **3-OS** | **CCRT** | **CCRT** | 1.08 (0.82, 1.42) | 0.95 (0.72, 1.27) | 1.2 (0.91, 1.59) | 1.14 (0.84, 1.55) | **5-OS** |
|  | **ICRT** | 0.89 (0.66, 1.21) | **ICRT** | 0.88 (0.64, 1.23) | 1.11 (0.86, 1.45) | 1.06 (0.74, 1.51) |  |
|  | **RT** | 0.97 (0.72, 1.3) | 1.08 (0.76, 1.55) | **RT** | 1.26 (0.91, 1.75) | 1.20 (0.84, 1.72) |  |
|  | **RT+S** | 0.78 (0.58, 1.03) | 0.87 (0.66, 1.14) | 0.8 (0.56, 1.14) | **RT+S** | 0.95 (0.70, 1.30) |  |
|  | **S** | 0.84 (0.61, 1.15) | 0.94 (0.63, 1.39) | 0.87 (0.59, 1.28) | 1.08 (0.76, 1.53) | **S** |  |
| **3-DFS** | **CCRT** | **CCRT** | 1.12 (0.85, 1.46) | 0.56 (0.4, 0.79) | 0.74 (0.27, 2.02) | 1.12 (0.20, 6.27) |  |
|  | **ICRT** | 1.06 (0.74, 1.52) | **ICRT** | 0.5 (0.32, 0.78) | 0.66 (0.25, 1.75) | 1 (0.18, 5.74) |  |
|  | **RT** | 1.31 (0.79, 2.16) | 1.23 (0.67, 2.29) | **RT** | 1.32 (0.45, 3.82) | 2 (0.35, 11.57) | **5-DFS** |
|  | **RT+S** | 1.63 (0.71, 3.77) | 1.54 (0.72, 3.28) | 1.24 (0.47, 3.31) | **RT+S** | 1.52 (0.21, 11.19) |  |
|  | **S** | 0.89 (0.25, 3.09) | 0.84 (0.23, 3.07) | 0.68 (0.18, 2.6) | 0.54 (0.12, 2.45) | **S** |  |
| **5-OSR** | **CCRT** | **CCRT** |  |  |  |  |  |
|  | **ICRT** | 1.05 (0.58, 1.9) | **ICRT** |  |  |  |  |
|  | **RT** | 1.92 (0.93, 4.14) | 1.8 (0.81, 4.26) | **RT** |  |  |  |
|  | **RT+S** | 0.74 (0.38, 1.55) | 0.7 (0.36, 1.45) | **0.39 (0.17, 0.9)** | **RT+S** |  |  |
|  | **S** | 0.6 (0.31, 1.17) | 0.57 (0.25, 1.3) | **0.31 (0.13, 0.74)** | 0.81 (0.37, 1.73) | **S** |  |

Abbreviation: S, surgery; RT, radiotherapy; RT+S, surgery combined with radiotherapy; ICRT, Induction chemotherapy radiotherapy; CCRT, current chemotherapy radiotherapy; 3-OS, 3-year overall survival; 5-OS, 5-year overall survival; 3-DFS, 3-year disease free survival; 5-DFS, 5-year disease free survival; 5-OSR, 5-year overall survival rate. The significant results were bolded.
