# Supplementary material for: Comparative efficacy of six therapies for Hypopharyngeal and laryngeal neoplasms: a network meta-analysis
Source: BMC Cancer. 2019 Mar 29;19:282. doi: 10.1186/s12885-019-5412-z (PMC6439970; doi:10.1186/s12885-019-5412-z)
Supplement: Supplementary file 3 — Table S3. SUCRA results of subgroup analysis for locally advanced hypopharyngeal and laryngeal neoplasms. (DOCX 16 kb) [file 12885_2019_5412_MOESM3_ESM.docx]

**Table S3.** SUCRA results of subgroup analysis for locally advanced hypopharyngeal and laryngeal neoplasms.

| **Treatment** | **3-OS** | **5-OS** | **3-DFS** | **5-DFS** | **5-OSR** |
| --- | --- | --- | --- | --- | --- |
| **CCRT** | 0.2046 | 0.3037 | **0.6947** | 0.5962 | 0.2004 |
| **ICRT** | 0.4904 | 0.5147 | 0.596 | **0.7707** | 0.1952 |
| **RT** | 0.3182 | 0.2138 | 0.3379 | 0.1319 | 0.0182 |
| **RT+S** | **0.8403** | **0.8082** | 0.2008 | 0.3779 | **0.2495** |
| **S** | **0.6465** | **0.6597** | **0.6706** | **0.6232** | **0.3366** |

Abbreviation: S, surgery; RT, radiotherapy; RT+S, surgery combined with radiotherapy; ICRT, Induction chemotherapy radiotherapy; CCRT, current chemotherapy radiotherapy; 3-OS, 3-year overall survival; 5-OS, 5-year overall survival; 3-DFS, 3-year disease free survival; 5-DFS, 5-year disease free survival; 5-OSR, 5-year overall survival rate. The top two SUCRA were bolded.
